# Elevated Urinary Biomarkers of Oxidative Damage in Photocopier Operators following Acute and Chronic Exposures

**DOI:** 10.3390/nano12040715

**Published:** 2022-02-21

**Authors:** Yipei Zhang, Anila Bello, David K. Ryan, Philip Demokritou, Dhimiter Bello

**Affiliations:** 1 Department of Chemistry, Kennedy College of Sciences, UMass Lowell, Lowell, MA 01854, USA; muerzhang@gmail.com (Y.Z.); david_ryan@uml.edu (D.K.R.); 2 Department of Public Health, Zuckerberg College of Health Sciences, UMass Lowell, Lowell, MA 01854, USA; anila_bello@uml.edu; 3 Department of Environmental Health, Harvard Center for Nanotechnology and Nanotoxicology, Harvard T.H. Chan School of Public Health, Boston, MA 02115, USA; pdemokri@hsph.harvard.edu; 4 Department of Biomedical and Nutritional Sciences, Zuckerberg College of Health Sciences, UMass Lowell, Lowell, MA 01854, USA

**Keywords:** oxidative stress, copier emitted nanoparticles, oxidative stress biomarkers, acute exposure, chronic exposure, reactive oxygen species, DNA damage, lipid peroxidation

## Abstract

Inhalation exposures to nanoparticles (NPs) from printers and photocopiers have been associated with upper airway and systemic inflammation, increased blood pressure, and cases of autoimmune and respiratory disorders. In this study we investigate oxidative stress induced by exposures to copier-emitted nanoparticles using a panel of urinary oxidative stress (OS) biomarkers representing DNA damage (8-hydroxydeoxyguanosine, 8-OHdG; 8-hydroxyguanosine, 8-OHG; 5-hydroxymethyl uracil 5-OHMeU), lipid peroxidation (8-isoprostane; 4-hydroxynonenal, HNE), and protein oxidation biomarkers (o-tyrosine, 3-chlorotyrosine, and 3-nitrotyrosine) under conditions of acute (single 6 h exposure, 9 volunteers, 110 urine samples) and chronic exposures (6 workers, 11 controls, 81 urine samples). Urinary biomarkers were quantified with liquid chromatography–tandem mass spectrometry after solid phase extraction sample cleanup. 8-OHdG, 8-OHG, 8-isoprostane, and HNE were significantly elevated in both the acute and chronic exposure study participants relative to the controls. In the acute exposure study, the geometric mean ratios post-/pre-exposure were 1.42, 1.10, 2.0, and 2.25, respectively. Urinary 8-OHG and HNE increased with time to at least 36 h post-exposure (post-/pre-exposure GM ratios increased to 3.94 and 2.33, respectively), suggesting slower generation and/or urinary excretion kinetics for these biomarkers. In chronically exposed operators, the GM ratios of urinary biomarkers relative to controls ranged from 1.52 to 2.94, depending on the biomarker. O-Tyrosine and 5-OHMeU biomarkers were not significantly different from the controls. 3-chlorotyrosine and 3-nitrotyrosine were not detected in the urine samples. We conclude that NPs from photocopiers induce systemic oxidative stress by damaging DNA, RNA, and lipids. Urinary levels of 8-OHdG, 8-OHG, HNE, and 8-isoprostane were orders of magnitude higher than in nanocomposite processing workers, comparable to nano titanium dioxide and fiberglass manufacturing workers, but much lower than in shipyard welding and carbon nanotube synthesis workers. Biomarkers 8-OHdG, 8-OHG, 8-isoprostane, and HNE appear to be more sensitive and robust urinary biomarkers for monitoring oxidative stress to NPs from photocopiers.

## 1. Introduction

Toner-based laser printing and photocopying is a multi-billion-dollar industry that is growing at an annual rate of 5.8% [1]. Engineered nanomaterials (ENMs), including zinc oxide, iron oxide, titanium dioxide, manganese oxide, copper oxide, aluminum oxide, amorphous silica, and occasionally ceria, have been incorporated into the toner formulation to improve printing quality [2,3,4]. It is now well-established that printing and photocopying results in the emission of high numbers of nanoparticles [5,6] that seem to be formed primarily from the condensation of semi-volatile organic compounds (SVOCs) evaporated from the toner during the printing process [7,8,9]. The daily geometric mean NP concentrations in copy centers in the Northeast USA ranged from 3700 to 34,000 particles/cm^3^, with emission peaks reaching up to 1.4 million particles/cm^3^ and a count median diameter centered around 30 nm [10,11]. Similar findings were reported recently in Singaporean photocopy centers [12] and in chamber studies [3]. The composition of the nanoscale fraction (PM_0.1_ or particulate matter less than 0.1 µm aerodynamic diameter) generated from the photocopiers has also been documented in three comprehensive studies [4,11,12] to include approximately 6–63% organic compounds (as organic carbon), <1% elemental carbon, and 2–8% metals. Polycyclic aromatic hydrocarbons (PAHs) species, including phenanthrene, fluoranthene, pyrene, chrysene, and benzo(b)fluoranthene at concentrations from 2–10 ng/m^3^ have been quantified in printer- and copier-emitted NPs [4,12,13]. Furthermore, toner-based printing and photocopying enrich airborne aerosols with higher molecular weight and more carcinogenic PAHs by approximately 2–3 fold [12,13,14]. More recently, we have documented that airborne nano aerosols from one toner-based printer produced short-lived free radicals and hydrogen peroxide, most of which originated from the 3% metal oxides present in these nanoparticles [14].

Toner-based laser printing and photocopying is a multi-billion-dollar industry that is growing at an annual rate of 5.8% [1]. Engineered nanomaterials (ENMs), including zinc oxide, iron oxide, titanium dioxide, manganese oxide, copper oxide, aluminum oxide, amorphous silica, and occasionally ceria, have been incorporated into the toner formulation to improve printing quality [2,3,4]. It is now well-established that printing and photocopying results in the emission of high numbers of nanoparticles [5,6] that seem to be formed primarily from the condensation of semi-volatile organic compounds (SVOCs) evaporated from the toner during the printing process [7,8,9]. The daily geometric mean NP concentrations in copy centers in the Northeast USA ranged from 3700 to 34,000 particles/cm^3^, with emission peaks reaching up to 1.4 million particles/cm^3^ and a count median diameter centered around 30 nm [10,11]. Similar findings were reported recently in Singaporean photocopy centers [12] and in chamber studies [3]. The composition of the nanoscale fraction (PM_0.1_ or particulate matter less than 0.1µm aerodynamic diameter) generated from the photocopiers has also been documented in three comprehensive studies [4,11,12] to include approximately 6–63% organic compounds (as organic carbon), <1% elemental carbon, and 2–8% metals. Polycyclic aromatic hydrocarbons (PAHs) species, including phenanthrene, fluoranthene, pyrene, chrysene, and benzo(b)fluoranthene at concentrations from 2–10 ng/m^3^ have been quantified in printer- and copier-emitted NPs [4,12,13]. Furthermore, toner-based printing and photocopying enrich airborne aerosols with higher molecular weight and more carcinogenic PAHs by approximately 2–3 fold [12,13,14]. More recently, we have documented that airborne nano aerosols from one toner-based printer produced short-lived free radicals and hydrogen peroxide, most of which originated from the 3% metal oxides present in these nanoparticles [14].

A series of in vitro and in vivo toxicological studies indicate that exposures to laser printer or photocopier emissions induce pulmonary inflammation, cytotoxicity, oxidative stress, and genotoxicity [15,16,17]. In our earlier studies, we investigated the kinetics of upper airway inflammation in a group of nine healthy volunteers following a single 6 h acute exposure over 36 h, as well as chronic inflammation following repeated exposures in six copier operators [18,19]. In the acute exposure study [18], nine healthy subjects spent 6 h at a busy photocopy center on two to three randomly selected days. They also spent one random day in an office environment with no NP exposures. Their urine and nasal lavage (NL) samples were collected before exposure (0 h), after exposure (6 h), and the next day after exposure (24 and 36 h). A notable increase 6 h after acute NP exposure was observed for several inflammatory cytokines in the NL, namely interlukin-6 (IL-6), interlukin-8 (IL-8), tumor necrosis factor α (TNFα), interlukin-1β (IL-1β), granulocyte-colony stimulating factor (G-CSF), epidermal growth factor (EGF), interlukin-10 (IL-10), monocyte chemoattractant protein-1 (MCP1), fractalkine, and vascular endothelial growth factor (VEGF). TNFα, IL-1β, G-CSF, IL-10, MCP1, and VEGF reached baseline levels within 24–36 h, whereas IL-6, IL-8, EGF, and fractalkine remained elevated even 30 h post-exposure. In chronically exposed copier operators, IL-6, IL-8, TNFα, IL-1β, and Eotaxin were significantly elevated in NL samples across different weeks, and week-to-week differences were not statistically significant. In addition, inflammatory PMN cell infiltration in NL was significantly increased (2.7-fold) compared with the control group [19]. 

Overproduction of ROS by printer- and copier-emitted nanoparticles can damage proteins, DNA, RNA, and lipids [20], resulting in oxidation products and damage-associated molecular patterns (DAMPs) products that trigger and/or sustain inflammation. The attack on polyunsaturated fatty acids in cell membranes by ROS, through free radical chain reactions, leads to lipid peroxidation. 8-isoprostane (or 8-isoprostaglandin F2_α_) is an important lipid peroxidation biomarker resulting from the non-enzymatic oxidation of arachidonic acid [21,22]. The oxidation of unsaturated fatty acids also produces several aldehydes in a cascade of breakdown by-products, which can be utilized as biomarkers of OS, including 4-hydroxynonenal (HNE), an unsaturated reactive aldehyde [21]. 8-hydroxydeoxyguanosine (8-OHdG), also known as 8-oxo-7,8-dihydro-2′-deoxyGuo (8-oxodG) and 8-hydroxyguanosine (8-OHG) (also known as 8-oxoGuo), are produced from the oxidation of guanine in the DNA and RNA chains, respectively, and are used as common biomarkers of DNA and RNA damage [23,24]. Another biomarker of oxidative DNA damage is 5-hydroxymethyl uracil (5-OHMeU) that results from thymine oxidation [25]. Three protein damage markers, namely o-tyrosine, 3-chlorotyrosine, and 3-nitrotyrosine, have been used as OS markers in other studies [26]. O-Tyrosine (o-Tyr) is an amino acid that is produced from the oxidation of phenylalanine by hydroxyl radicals [27]. Because it does not occur naturally, o-Tyr can serve as a specific biomarker of protein oxidation by hydroxyl radicals. 3-Chlorotyrosine is the oxidation product of p-tyrosine by the highly reactive hypochlorous acid (oxidation product of myeloperoxidase), while 3-nitrotyrosine is produced by the nitration of p-tyrosine, mediated by reactive nitrogen species, including peroxynitrite anion and nitric oxide [27]. 

In our previously mentioned studies on copier operators [18,19], we also measured 8-OHdG in urine with an ELISA kit and found a 2- to 10-fold post-exposure increase in acutely exposed healthy volunteers and 4.3-fold increase in chronically exposed operators relative to the controls. Because ELISA-based colorimetric assays suffer from potential interferences and overestimation of 8-OHdG [28,29], liquid chromatography–tandem mass spectrometry is preferred as a more accurate and specific method, which can also measure additional OS biomarkers. In this study, we investigated a panel of urinary biomarkers of OS in two groups of individuals—health volunteers exposed once to copier emitted nanoparticles [18] and chronically exposed workers [19]. The panel of urinary OS biomarkers in vivo included 8-OHdG, 8-OHG, 5-OHMeU, o-tyrosine, 8-isoprostane HNE, 3-chlorotyrosine, and 3-nitrotyrosine. We found significantly elevated levels of 8-OHdG, 8-OHG, 8-isoprostane, and HNE in both healthy volunteers and chronically exposed workers. The findings of this study confirmed our earlier observation of elevated urinary levels of 8-OHdG measured by ELISA and further strengthens the argument that copier-emitted nanoparticles do induce significant levels of OS in humans. In this paper, we refer exclusively to toner-based printing and photocopying, unless otherwise noted. For simplicity and consistency, we will refer to printer- and copier-emitted nanoparticles in copy centers as CNPs.

## 2. Materials and Methods

### 2.1. Study Design

#### 2.1.1. Acute CNP Exposure Study Design

Details of the study design have been previously reported by our group [18]. Briefly, in the acute exposure study, nine young, healthy, and non-smoking volunteers were asked to spend 5–6 h/day in a copy center. Five subjects volunteered to repeat the experiment three times, whereas four volunteers only two times, two–three times, in random non-consecutive days, weeks to months apart. Each volunteer gave NL and urine samples before exposure (U_0_), at the end of the exposure period (U_6_), the morning the next day (U_24_), and the next day at the end of their shift (U_30_). The same volunteers were also asked to spend an equal amount of time (6 h) for a single day in an office environment with no photocopying activities. Urine and NL samples were also collected from these controls at the beginning and end of the exposure period using the same sampling protocol. Each volunteer donated 8–12 samples (4 samples/day × 2 or 3 days; n = 92 urine samples) as part of the controlled CNP exposure, and another 2 urine samples (2 × 1 × 9; n = 18) as part of the background exposure day. A total of 110 urine samples were analyzed.

#### 2.1.2. Chronic CNP Exposure Study Design

The study design was previously reported by our group [19]. Briefly, six full-time copier operators from three commercial centers, whose primary job was printing and photocopying, were recruited for the study. Each operator was sampled for two–three random weeks over the course of two years in order to assess the variability of NP exposure and the effects on biomarkers. Eleven individuals who were not involved with any printing and photocopying activities also participated in this study as the matched control group. Urine samples were collected on a Monday morning pre-shift (Mo-AM) and post-shift (Mo-PM), as well as at the end of the workweek (Friday post-shift or Fr-PM). Individuals in the control group were asked to give urine samples over the course of the study in accordance with the same protocol. Five copier operators donated nine urine samples over three weeks, whereas one subject participated for only one week (5 subjects × 3 samples/week × 3 study weeks + 1 × 3, n = 48 urine samples). The eleven controls donated three urine samples (11 subjects × 3 samples/week, n = 33 samples) urine samples. A total of 81 urine samples were analyzed as part of this component of the study.

#### 2.1.3. Urine Sample Collection

Urine samples were collected for both the acute and chronic CNP exposure studies immediately after each exposure period and centrifuged at 5000× *g* for 10 min at 4 °C. Samples were then aliquoted into several individual 2 mL polypropylene cryovials, spiked with 100 μg of 3,5-Di-*tert*-4-butylhydroxytoluene (BHT) antioxidant, and stored at −80 °C until analysis. 

All subjects gave their informed consent for inclusion before they participated in the study. The study was conducted in accordance with the Declaration of Helsinki, and the protocol was approved by the institutional review board of the University of Massachusetts Lowell.

### 2.2. Exposure Characterization 

Detailed exposure characterization was conducted for both studies and has been reported in detail in our earlier work by Bello et al. and Khatri et al. [4,11,18,19]. Exposure characterization included real-time nanoparticle exposure data, number concentration, mass size distribution, extensive physico-chemical exposure characterization of nanoparticles, as well as monitoring for gaseous co-pollutants such as ozone, carbon monoxide, carbon dioxide, and total volatile organic compounds.

### 2.3. Sample Preparation for LC-ESI-MS/MS Analysis 

#### 2.3.1. Chemicals and Materials 

Both 8-hydroxy-2′–deoxyguanosine (purity ≥ 98%) and 5-hydroxymethyluracil (purity 97%) were purchased from Sigma-Aldrich (St. Louis, MO, USA), and 8-hydroxy-2′-deoxyguanosine-15N5 (purity 95%) was purchased from Cambridge Isotope Laboratories, Inc. (Tewksbury, MA, USA). 8-hydroxyguanosine (purity ≥ 98%), 8-isoprostane (purity ≥ 99%), and 8-isoprostane-d4 (purity ≥ 99%) were obtained from Cayman Chemical (Ann Arbor, MI, USA). 8-hydroxyguanosine-13C,15N2 (purity 98%), 4-hydroxynonenal (purity ≥ 98%), and 4-hydroxynonenal-d3 (purity 97%) were purchased from Santa Cruz Biotechnology (Dallas, TX, USA). Methanol (LC-MS grade) was purchased from Burdick and Jackson (Muskegon, MI, USA). Formic acid and ammonium hydroxide were obtained from Sigma-Aldrich (St. Louis, MO, USA). Strata-X-A cartridges (33 µm Polymeric Strong Anion, 200 mg/3 mL; 8B-S123-FBJ), analytical columns (Kinetex phenyl-hexyl column, 100 mm × 4.6 mm, 2.6 µm particle size; 00D-4495-E0), and Kinetex C18 column, (100 mm × 4.6 mm, 2.6 μm particle size, 00D-4462-E0) were purchased from Phenomenex (Torrance, CA, USA). 

#### 2.3.2. Urine Processing and Cleanup Using Solid-Phase Extraction (SPE) 

Sample processing and cleanup was conducted before LC-ESI-MS/MS analysis. A detailed description of the sample preparation method is provided in Appendix A. Briefly, a 1 mL aliquot of the urine sample was thawed at room temperature and spiked with an internal standard (IS) cocktail yielding a concentration of 50 ng/mL each of 3-Nitro-L-tyrosine-13C6, 8-OHdG-15N5, 3-Chloro-L-tyrosine-13C6, L-tyrosine-d4, 8-OHG-13C15N2, and 8-isoprostane-d4. For HNE analysis, which required derivatization, a separate 0.5 mL urine aliquot was spiked with 4HNE-d3 to give 10 ng/mL IS and processed separately as described below. The urine samples were subsequently vortexed, and the protein was precipitated by the addition of 2 mL of cold acetone and stored for 30 min at 4 °C. The resultant suspension mix was centrifuged at 3000 rpm for 15 min. The supernatant was transferred into a new vial, evaporated to dryness in a vacuum oven, and reconstituted to 1 mL with 5% ammonium hydroxide in water. The reconstituted samples were subsequently precleaned with Strata-X-A SPE cartridges. The SPE method is detailed in Appendix A. The first 1.0 mL urine sample yielded two fractions after SPE cleanup: Fraction A, which contained biomarkers of DNA and protein oxidation, and Fraction B, which contained 8-isoprostane and similar compounds. The second urine aliquot of 0.5 mL yielded a third fraction, C, and contained HNE. HNE in Fraction C was derivatized by adding 2,4-dinitrophenylhydrazine (DNPH) to convert it to the stable analyte of HNE-DNPH. The derivatization protocol was based on a previous publication with slight modifications [28]. Briefly, 10 µL of DNPH solution (0.05 M in acetonitrile and acetic acid 9:1, *v*/*v*) was added into the sample eluate and the standards, and they were placed in a water bath at 40 °C for 2 h. Then the solution was dried under vacuum and reconstituted in 200 µL of acetonitrile. Three fractions were analyzed separately with LC-ESI-MS/MS as described below. Fraction A contained small polar compounds that were best separated on a phenyl-hexyl column, and they were also more sensitive in positive ionization mode. Fraction B and C contained larger lipid molecules that separated well on a C18 column and were more sensitive in the negative ionization mode.

#### 2.3.3. LC-ESI-MS/MS Analysis—Apparatus and Conditions 

All urine samples were analyzed using a Shimadzu LC-20AD chromatographic system coupled with an API 3200 triple quadruple mass spectrometer equipped with a Turbo Ion Spray source (Applied Biosystems, Foster City, CA, USA). Details of the chromatographic system, chromatographic conditions (column, flow rates, mobile phases, etc.), compound specific multiple reaction monitoring MRM method setup, and calibration curves information are presented in Appendix A and in Table A1. Standard calibration curves containing 8-OHdG, 8-OHG, O-tyrosine, 8-isoprostane, 5-OHMeU, and HNE-DNPH in the 0.25 to 500 ng/mL range were prepared in 30% methanol in water. The standard solutions were spiked with 10 ng of their corresponding internal standards (8-OHdG-15N5, 8-OHG-13C,15N2, L-tyrosine-d4, 8-isoprostane-d4, and HNE-d3-DNPH). No commercial isotopically labeled IS for 5-OHMeU is available. L-tyrosine-d4 was used as an IS for 5-OHMeU because of similar retention times. Quantitation was based on the IS method. The LC-ESI-MS/MS method was thoroughly validated as detailed in Appendix A by assessing the following parameters: accuracy, precision, sensitivity, recovery, calibration curve performance, and process efficiency. Linearity, sensitivity, and reproducibility were excellent, as was the recovery of all analytes. Matrix effects were also investigated and found to be negligible. Analyte specific LODs were in the range from 50–1000 pg/mL (Table 1). 

#### 2.3.4. LC-ESI-MS/MS Analysis of Creatinine 

The protocol for the LC-ESI-MS/MS of creatinine was based on the previously published literature [30]. Briefly, the thawed urine samples were diluted first by 100× (10 µL into 990 µL DI water), followed by a second 20× dilution (50 µL into 1 mL final) in LC amber glass vials. Ten nanogram of creatine-d3 were spiked into this second LC vial (final concentration, 10 ng/mL). This urine solution was subject to LC-ESI-MS/MS analysis [30]. 

### 2.4. Statistical Analysis 

Urinary biomarkers were normalized to creatinine to adjust for variations in urinary dilution and were expressed as nanogram biomarker/picomole creatinine. Biomarker data were examined for the underlying distributions using the Shapiro–Wilks statistics and by graphing probability plots and histograms using the SAS System for the PC (SAS v 9.2 Inc., Carry, NC, USA). Urinary biomarker data were found to be lognormally distributed and all subsequent analyses were performed on log-transformed data. Biomarkers below the limit of detection were estimated as LOD/√2, when they represented less than 25% of the total samples. Because of the repeated measurement design, mixed models with a random intercept and compound symmetry covariance structure were used to test for cross-day and cross-week changes in urinary biomarker levels. Paired t-tests were performed on log-transformed measurements to test for the differences in biomarkers between background morning (U_0_) and afternoon (U_6_) samples. An unpaired t-test was performed for the biomarker differences between the control subjects exposed to background particles and CNPs. The relationships between the OS biomarkers in the urine of acute exposure study volunteers were evaluated by multiple regression analysis. Graphs were prepared in SAS and/or in GraphPad Prism 7.00. *p* values were considered significant if *p* < 0.05. Exposure data, their distributions and summary statistics, have been described in detail elsewhere [11,18,19].

## 3. Results

### 3.1. Urine Processing and Cleanup Using SPE 

Urine is a complex biological matrix that contains urea, inorganic salts, protein, and over one thousand organic compounds. Solid-phase extraction is a widely used sample preparation technique for cleaning and concentrating biological samples that offers higher sample purity and better recovery and reproducibility compared to liquid–liquid extraction. In previously published literature, a C18 reverse phase SPE was used to isolate biomarkers of oxidative stress in different types of biofluids [24,25]. In this study, however, the C18 cartridge provided poor retention for DNA and protein damage markers (8-OHdG, 8-OHG, o-tyrosine, 5-OHMeU), which are small and relatively polar compounds. Based on the pKa of these analytes (pKa, 4.36 to 7.55), all of them, except HNE, can be negatively charged under alkaline conditions. Therefore, a mix-mode polymeric SPE cartridge (Strata^TM^-X-A, strong anion, Phenomenex) was utilized for their retention in this application. The process efficiency and reproducibility of the SPE procedure using Strata-X-A, are summarized in Table 1. Mean recoveries of all analytes, except 5-OHMeU, were in the 90–98% range. 5-OHMeU had the lowest recovery with 86%. Precision, which ranged from 5.4% RSD to 12.3% RSD, was excellent for all analytes. HNE and 8-isoprostane had the highest process efficiency, i.e., 94.5% and 95.6% respectively, due to their strong hydrophobic interaction with the SPE cartridge sorbent. For relatively polar compounds, including 8-OHdG, 8-OHG, o-tyrosine, and 5-OHMeU, the process efficiency varied from 76.5% to 89.5%. 

### 3.2. LC-ESI-MS/MS Analysis 

Linear regression equations of the calibration curves for each biomarker over the tested range of 50 pg/mL to 1 µg/mL are summarized in Appendix A, Table A2. The calibration curves were linear in the tested range with the correlation coefficient R^2^ ≥ 0.999. The limit of detection (LOD) was determined based on the instrument response with the integrated function of the Analyst 1.4.2 software (Applied Biosystems). These calculations were based on signal/noise ratios of 3 and 10 for LOD and LOQ, respectively. Table 1 is a summary of the LOD/LOQ of analytes. HNE-DNPH had the highest sensitivity (LOD, 0.05 ng/mL). The LOD for all analytes ranged from 0.25 ng/mL to 1 ng/mL. The lowest standards in the calibration curve had a reproducible signal-to-noise ratio of 6, which is above the FDA definition of LLOQ (lower limit of quantification), defined as ≥ five times the analyte response of the zero calibrator (S/N 5). The concentration of all analytes in the urine samples after SPE treatment were above their LOD with most of the analytes being above their LOQ. However, the concentration of 8-isoprostane in the urine was relatively low, from 0.75 ng/mL to 21.3 ng/mL, resulting in approximately 10% of the urine samples below the respective LOQ. In this study, two protein oxidation biomarkers in urine the samples, 3-chlorotyrosine and 3-nitrotyrosine, were below their limit of detection (0.5 ng/mL) and they have been omitted from subsequent presentations and discussion. 

### 3.3. Acute Exposure Study: Urinary OS Biomarker Concentrations

**Figure 1:**
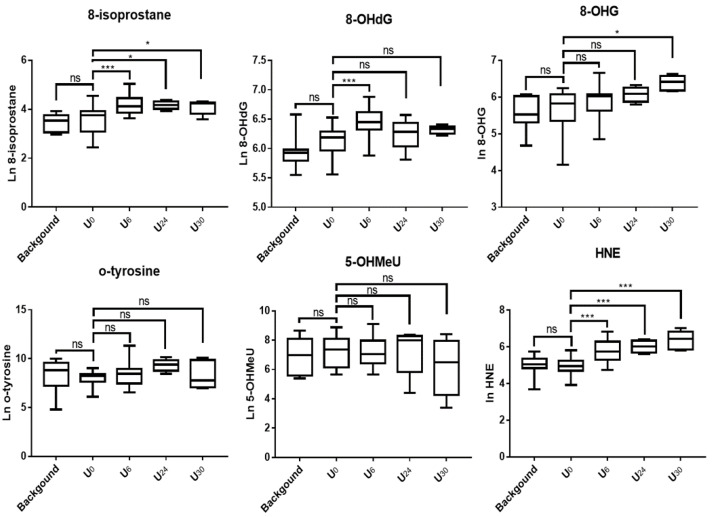
Box plot distribution of urinary OS biomarkers of nine healthy volunteers following a 6-h acute exposure episode. Legend: ns, not statistically significant; * (*p* < 0.05); and *** (*p* < 0.001). Background, average of AM and PM values in a non-exposure day; U_0_, pre-exposure; U_6_, end of single 6-h exposure episode; U_24_, next day morning; U_36_, next day afternoon.

Table 2 and Figure 1 summarize urinary biomarker values for healthy volunteers following a single acute exposure of 5–6 h in a photocopy center at various time points (U_0_, prior to exposure; U_6_, immediately post exposure; U_24_, next day AM; U_30_, next day PM), as well as urinary background values of the same subjects in the morning (AM) and afternoon (PM) of the day they spent in an adjacent office environment free of CNPs. The background biomarker values were not significantly different between AM and PM (8-isoprostane, *p* = 0.94; 8-OHdG, *p* = 0.51; 8-OHG, *p* = 0.55, o-tyrosine, *p* = 0.44; 5-OHMeU, *p* = 0.68; HNE, *p* = 0.80). Furthermore, they were not significantly different from the pre-exposure U_0_ values. 

In the acute exposure study, 8-OHdG, 8-isoprostane, and HNE levels were higher and statistically significantly different in samples immediately after exposure (U_6_) relative to pre-exposure samples (U_0_). The GM concentration of 8-OHdG in pre-exposure samples was 441.9 ng/pmol creatinine and increased to 627.5 ng/pmol creatinine (*p* < 0.001) immediately post-exposure. The ratio of the (post-exposure/pre-exposure) geometric mean values of 8-OHdG was 1.42. By the next morning (U_24_), the 8-OHdG concentration decreased to 515 ng/pmol creatinine (U_24_) and 555.4 (U_30_) and were not significantly different from pre-exposure levels and the controls. The shape of the 8-OHdG curve was similar to that reported earlier in Khatri et al., 2013 [18]. The GM concentration of 8-OHG for pre-exposure was 253.4 ng/pmol. No significant increases in 8-OHG were seen post-exposure (280.1 ng/pmol) relative to pre-exposure and the control. An upward trend in urinary 8-OHG was observed post-exposure, and 8-OHG at U_30_ (592.2 ng/pmol) was significantly higher than pre-exposure and the control samples (*p* < 0.05). The U_30_/U_0_ GM ratio was 2.33.

The concentration of HNE continued to increase post-exposure at least until U_30_. The GM concentrations of HNE at U_0_, U_6_, U_24_, and U_30_ were (ng/pmol): 147.6, 332.4, 409.7, and 581.9 ng/pmol, respectively. The ratio of U_6_/U_0_ for HNE was 2.25, and HNE in U_6_ was significantly higher than in U_0_ (*p* < 0.001). No statistically significant differences were found between U_6_, U_24_, and U_30_. The GM ratio of U_30_/U_0_ was 3.94. The concentrations of 8-isoporstane were amongst the lowest of all biomarkers: GM, 32.29 ng/pmol (U_0_), 65.09 ng/pmol (U_6_), 64.67 ng/pmol (U_24_), and 59.55 ng/pmol (U_30_). The GM ratio of U_6_/U_0_ was 2.02, and U_6_ was significantly higher than U_0_ (*p* < 0.001). The level of 8-isoprostane decreased only slightly at U_30_ (next day PM), or 24-h post-exposure (GM ratios of U_30_/U_0_ = 1.84). 

O-tyrosine and 5-OHMeU concentrations did not change from the background and were not statistically significantly different (Figure 1, Table 2).

### 3.4. Chronically Exposed Photocopier Operators: Urinary OS Biomarker Concentration

**Figure 2:**
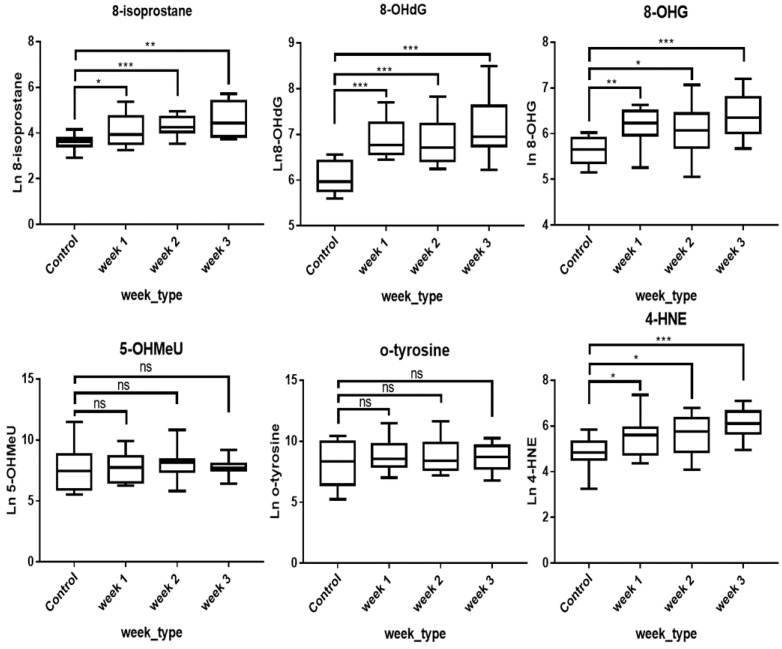
Box plot distribution of OS biomarkers in urine from the chronic exposure study. ns, not statistically significant, * (*p* < 0.05), ** (*p* < 0.01), and *** (*p* < 0.001).

In the chronic exposure study, four of the six biomarkers, namely 8-OHdG (*p* < 0.001), 8-OHG (*p* < 0.05), HNE (*p* < 0.05), and 8-isoprostane (*p* < 0.05), were significantly higher than in the control group (Table 3, Figure 2). There were no statistically significant differences in the mean levels of these biomarkers between different weeks (week 1, week 2, and week 3) (Table 4). The ratio of GM concentrations of the urinary biomarkers in the chronically exposed operators relative to the controls ranged from 1.52 to 2.94, depending on the biomarker. Generally, the GM concentrations of 8-isoprostane, HNE, 8-OHdG, and 8-OHG in the chronic exposure group were higher than the acute exposure group (U_6_). The ratio of GM concentrations of 8-isoprostane, HNE, 8-OHG, and 8-OHdG in the chronic exposure setting relative to U_6_ of acute exposure was 1.46, 1.32, 1.99, and 1.87, respectively. As illustrated in Figure 2, the mean 8-OHdG (*p* < 0.001) and 8-OHG (*p* < 0.05) was significantly higher in the copier operators relative to the acute exposure group at U_6_. Similar to the acute study, o-tyrosine and 5-OHMeU were not elevated and not statistically different from the controls. 

### 3.5. Correlation between Urinary OS Markers

Several OS biomarkers were found to be correlated with each other in the urine of the acutely exposed volunteers immediately post-exposure (U_6_) and one day after acute exposure (U_30_) samples, as shown in Table 5. In the U_6_ samples, 8-isoprostane was correlated moderately with 8-OHdG (Spearman, 0.54; *p* < 0.05) and HNE (Spearman, 0.59; *p* < 0.01), but weekly with 8-OHG (Spearman, 0.32; *p* < 0.05). In addition, 8-OHdG was correlated strongly with 8-OHG (Spearman, 0.72; *p* < 0.001) and moderately with HNE (Spearman, 0.45; *p* < 0.05). Similarly, in the U_30_ urine samples, 8-isoprostane was correlated with 8-OHdG (*p* < 0.05) and HNE (*p* < 0.05) (data omitted).

Similar correlations were observed in the urine of the chronically exposed copier operators. 8-OHdG was correlated with HNE (Spearman, 0.33; *p* < 0.05) and 8-OHG (Spearman, 0.35; *p* < 0.05)

## 4. Discussion

Several human epidemiological studies have documented that inhalation exposure to printer and copier emitted nanoparticles induce oxidative stress. Kleinsorge et al. [31] documented significantly increased levels of lipid peroxidation (TBARS) in full-time copier operators. Elango et al. [32] found elevated plasma 8-isoprostane and serum TBARS in copier operators accompanied by a reduction in the serum total ferric reducing antioxidant capacity. Another study by Könczöl et al. [33] showed an overproduction of ROS in human epithelial A549 lung cells after cell exposure to printer emitted particles. An earlier study by our group [34] showed that the dosing of induced human THP-1 cells with varying administered doses (30–300 µg/mL) of photocopier-emitted nanoparticles resulted in the up-regulation of oxidative stress genes (HO1). 

Urinary OS biomarkers of DNA/RNA and lipid peroxidation increased several-fold after a single exposure and were even higher in chronically exposed copier operators. Although many studies reporting results of these oxidative damage markers in urine exist (see later discussion), little is known about the clearance kinetics of these markers in urine. In this regard, our time course study of healthy volunteers exposed acutely to CNP provide some important insights. In the acute exposure study, maximum values of 8-OHdG and 8-isoprostane were observed shortly after the end of the six-hour exposure suggesting relatively fast production rates in tissues (likely the airways) and clearance rates in urine. Furthermore, their urinary levels remained relative steady up to 30 h. In contrast, 8-OHG and HNE reached the highest observed values at 30 h (24-h post-exposure), the last studied timepoint, and it is likely that their true maximum may have not been observed. This suggests slower urinary clearance kinetics for these biomarkers. Another factor to consider is that nanoparticles deposited in the deep lungs are cleared slowly and, as a result, may continue to induce OS [15,35,36]. 

Oxidative damage of RNA, reflected in elevated urinary 8-OHG, especially the oxidation of messenger RNA (mRNA), reduces the efficiency of translation during protein synthesis, which may further lead to protein mutations and ultimately cell death [37,38]. 8-isoprostane, in comparison with other lipid peroxidation biomarkers (mostly reactive aldehydes), is a more chemically stable and a reliable biomarker of lipid peroxidation that can be used to assess the status of oxidative stress [22,39]. Lipid peroxidation may induce membrane damage, inactivate enzymes and proteins, and produce damage-associated molecular patterns (DAMPs), leading to further inflammation and cardiovascular disease [39]. 

O-tyrosine is formed primarily from the oxidation of the benzyl ring of phenylalanine by hydroxyl radicals. Elevated levels of o-tyrosine in biofluids have also been observed in patients with cataracts and type II diabetes [40]. In this study, we did not find elevated levels of o-Tyrosine in CNP exposed volunteers or operators relative to pre-exposure or the controls. 3-Chlorotyrosine and 3-nitrotyrosine were not detected. More sensitive instrumentation and/or much larger urine volumes (5 mL or more) may be needed to detect these biomarkers. Their absence suggests that the mechanisms of their formation—reactive nitrogen species and nitric oxide for 3-nitrotyrosine and hypochlorous acid oxidation from myeloperoxidase in neutrophils for 3-chlorotyrosine—may not be predominant mechanisms for CNPs. 

It is important to place these findings in the context of copier-emitted nanoparticle exposures, which have been well-characterized in this study. In the acute exposure study, the daily geometric mean concentration was in the range from 20,000 to 30,000 particles/cm^3^ [18]. In the chronic exposure study, the daily geometric mean total particle number concentration varied between 14,600 and 21,860 particles/cm^3^ [19]. The Multiple Particle Path Dosimetry Model software (MPPD v.3) estimated total particle deposition in the lungs to range from 28% to 40%, of which 5–7% deposit in the head airways, 7–13% in the thoracic region, and 14–20% in the alveolar space [2,11]. Although engineered nanomaterials (metals or metal oxides) only accounted for ~2–8% total mass of nanoparticles [2,11], they play an important role in generation of ROS. Transition metals and their metal oxides in these nanoparticles, which include Mn, Cr, Co, Cu, and Ni, can facilitate Fenton reactions to form highly reactive hydroxyl radicals from hydrogen peroxide, leading to DNA and RNA damage and lipid peroxidation. It is highly likely that the organic fraction also contributes to OS. In an earlier study, we quantified ROS generated from the organic fraction of printer emitted particles. The formation of short-lived ROS and H_2_O_2_ induced by the organic fraction was determined to be 0.038 nmol H_2_O_2_ eq./µg and 0.19 nmol/µg, respectively. However, the majority of ROS and H_2_O_2_ was formed by the 3% metal/metal oxide content in the nanoscale fraction. The organic fraction may also contain redox cycling organic compounds which can contribute to ROS formation. In past studies, several high molecular weight PAHs were identified in the organic fraction of photocopier- and laser printer-emitted particles including chrysene, benzo[a]anthracene, benzo[b/j]fluoranthene, and benzo[k]fluoranthene [14]. These may be derived from the low molecular weight PAHs which are present in toners, and they may be metabolically converted to redox-active quinones [41]. These quinones can generate superoxide anion from molecular oxygen, further contributing to OS damage. 

There are no biological exposure indexes or recommended guidance values for urinary OS markers against which to compare our current findings. Therefore, a comparison with the existing occupational health and toxicology literature will be helpful. Table 6 (references therein) provides a relevant summary of existing studies. The OHdG AM value in our small group of workers was ~3.5 times higher than in a healthy population; 2 times higher than in the Singaporean copier operators; comparable to, albeit at the lower half the nano TiO_2_ exposed workers, much higher than in the nanocomposite synthesis and processing workers, and ~4 times lower than in the workers handling and manufacturing carbon nanotubes and metal oxides (Table 6). 

8OHG arithmetic mean values were much higher (12 times) than in the nanocomposite workers, comparable to painters and gasoline attendants, but much lower than in the fiberglass and TiO_2_ workers. 5OHMeU values in our groups of subjects was not statistically significantly different from the controls. The absolute AM values of 5OHMeU, however, were over 500 times higher than in the nanocomposite workers; and over 60 times higher than in the Singaporean cohort. 

The lipid oxidation marker HNE was comparable to the Singaporean copier operators (Table 6, our own unpublished data). Likewise, HNE levels were comparable to the Singaporean copier operators. 8-Isoprostane AM was 23 times higher than in the nanocomposite workers, and 3 times higher than in the Singaporean copier operators. O-tyrosine AM values of in this study were 20 times higher than in nanocomposite works; and 7.2 times higher than in the Singaporean cohort. 

## 5. Conclusions

In this study, a sensitive and selective LC-ESI/MS/MS-based analytical method was developed to quantify several oxidative stress biomarkers in urine from two aliquots of 1.0 and 0.5 mL of the samples, respectively. An SPE pre-treatment procedure was developed to purify the sample and concentrate the analytes, resulting in increased sensitivity and improved method performance for routine use in widely available LC-MS systems. This method was evaluated extensively and proved to be sensitive, precise, and accurate. The level of 8-OHdG, 8-isoprostane, and HNE were significantly elevated in the urine of the healthy volunteers after acute exposure, as well as in the chronically exposed photocopier operators. Interestingly, 8-OHG and HNE in the urine of the acutely exposed healthy volunteers increased with time to at least 36 h post-exposure, suggesting slower excretion kinetics for these biomarkers relative to other biomarkers, such as 8-OHdG. Mean 8-OhdG in the urine of the copier operators was ~2.6 times higher than in the controls, and comparable to the lower tercile of the nano TiO_2_ manufacturing workers reported in previous studies. Similarly, mean 8-isoprostane was 2.9 times higher than in the controls, 23 times higher than in the nanocomposite workers, and 29 times lower than in the shipyard welders. Overall, our findings confirm that NPs from photocopiers induce systemic oxidative stress, leading to DNA (8-OHdG), RNA (8-OHG), and lipid (HNE, 8-isoprostane) oxidation, as well as upper airway inflammation, as documented in earlier work. 8-OHdG, 8-OHG, 8-isoprostane, and HNE appear to be more sensitive and robust urinary biomarkers for monitoring oxidative stress to NPs from photocopiers. Further research is needed to document the role of systemic oxidative stress and inflammation in the development of respiratory and cardiovascular disease in these workers.

## Figures and Tables

**Table 1:** Method validation for the set of OS markers in urine.

Biomarker	Lowest Standard Concentration in the Calibration Curve (ng/mL) (S/N = 6) ^a^	LOD (ng/mL) S/N = 3	LOQ (ng/mL) S/N = 10	Precision RSD (%)	Accuracy (%)	Mean Recovery (%)	Process Efficiency (%)
8-OHdG	0.50	0.25	0.75	5.8	−8.8	91.2 ± 5.1	79.8
8-OHG	1.00	0.50	1.50	9.7	−6.5	93.5 ± 6.8	89.5
5-OHMeU	2.00	1.00	3.00	8.0	−13.5	86.5 ± 4.5	78.5
8-isoprostane	1.00	0.50	1.50	5.4	−3.5	96.5 ± 7.7	95.6
HNE	0.10	0.05	0.15	7.4	−2.7	97.3 ± 4.3	94.5
o-Tyrosine	1.00	0.50	1.50	12.3	−10.8	89.2 ± 6.6	76.5

^a^ Lowest standard in the curve was selected to yield a S/N ratio of 6, which was above the FDA’s lower limit of quantitation (LLOQ), defined as ≥ five times the analyte response of the zero calibrator (S/N = 5).

**Table 2:** Summary statistics on the oxidative stress biomarkers in urine of nine volunteers on background (one day/subject, n = 18) and exposure days (2–3 days/subject, n = 92). Data represents the geometric mean (ng/pmol creatinine) and 95% confidence interval of the mean. U_AM_, morning urine; U_PM_, afternoon urine; U_0_, pre-exposure; U_6_, end of single 6-hr exposure episode; U_24_, next day morning; U_36_, next day afternoon.

Biomarkers (ng/pmol Creatinine)		Background, Non-Exposure Day		Acute CNP Exposure Day			
		U_AM_	U_PM_	U_0_	U_6_	U_24_	U_36_
8-OHdG	GM (Range)	404.5 (281.2, 581.9)	353.8 (305.5, 405.8)	441.9 (388.8, 502.1)	627.5 (556.6, 707.4)	515 (366.9, 722.9)	555.4 (510, 604.9)
8-OHG	GM (Range)	272.2 (152.1, 487.2)	232.5 (177.1, 305.3)	253.4 (190.6, 336.9)	280.1 (214.9, 365.2)	368.9 (301.9, 450.8)	592.2 (460.4, 761.7)
5-OHMeU	GM (Range)	1237 (306.9, 4988)	1284 (272.7, 6046)	1456 (885.4, 2394)	1352 (844, 2384)	1943 (595.4, 6334)	944.1 (205.5, 4338)
8-Isoprostane	GM (Range)	32.5 (21.21, 49.81)	31.72 (23.8, 42.28)	32.29 (26.29, 42.15)	65.09 (54.54, 77.68)	64.67 (52.43, 79.76)	59.55 (41.44 85.57)
HNE	GM (Range)	154.2 (103.4, 230)	138.2 (52.75, 362.1)	147.6 (115.7, 188.1)	332.4 (247.2, 446.9)	409.7 (269.9, 621.9)	581.9 (308, 1100)
o-Tyrosine	GM (Range)	2572 (414.5, 15,960)	3583 (422.7, 29,004)	2754 (1804, 4205)	3326 (2346, 4716)	4881 (2669, 8926)	3709 (1280, 10,749)

**Table 3:** Summary statistics of OS biomarker in the urine of eleven controls (33 urine samples) and six chronically exposed copier operators (6 operators, 48 urine samples) averaged over the three study weeks. Data represents the geometric mean (ng/pmol creatinine), 95% confidence interval of the mean, and minimum and maximum value. *p*-value represents *t*-test for mean differences.

Biomarkers	Controls		Chronic Exposure (Three Weeks)		*p* Value
	Mean (95% CL)	Min–Max	Mean (95% CL)	Min–Max	
8-OHdG	447 (336.9, 557.2)	271.2–702.7	1175 (952.3, 1398)	502.7–2900	0.0010
8-OHG	366.6 (240, 493.2)	173.4–638.6	556.1 (462.2, 650)	156.9–1136	0.0405
5-OHMeU	3065 (1858, 5056)	794.7–7366	2144 (1615, 2847)	334.8- 9796	0.2605
8-Isoprostane	38.05 (29.27, 46.8)	18.63–63.86	95.01 (71.57, 118.5)	25.79–304.7	0.0093
HNE	149 (84.68, 213.4)	25.82–342.6	438.1 (314.1, 562)	59.6–1567	0.0153
o-Tyrosine	3566 (1886, 6745)	553.1–10,403	3775 (3054, 4667)	887.1–8780	0.6935

**Table 4:** Summary statistics of OS biomarker in urine of six chronically exposed copier operators (48 urine samples) over three random weeks. Data represents the geometric mean (ng/pmol creatinine), 95% confidence interval of the mean, minimum value, and maximum value.

Biomarker	Week 1		Week 2		Week 3		*p* Value
	Mean	Min–Max	Mean	Min–Max	Mean	Min–Max	
8-OHdG	1185 (711.7, 1409)	630.8–2196	1060 (711.7, 1409)	515–2509	1302 (788.4, 1816)	502.7–2900	0.6655
8-OHG	511.4 (388.4, 634.5)	192.3–759.7	485.6 (308.2, 662.9)	156.9–1172	673.6 (473.4, 897.8)	291.4–1336	0.1950
5-OHMeU	2286 (1178, 4438)	528.9–8377	2113 (1295, 3448)	334.8–5334	2063 (1286, 3310)	611.2–9796	0.8349
8-Isoprostane	78.72 (35.83, 121.6)	25.79–214.3	82.19 (61.8, 121.6)	41.51–142.4	126.7 (62.38, 191)	46.07–304.7	0.1790
HNE	397.9 (91.67, 704.1)	77.77–1567	420.5 (203.3, 637.8)	59.6–1206	495.4 (292.9–697.8)	141.9–1206	0.8098
o-Tyrosine	3835 (2488, 5910)	1122–7604	3580 (2339, 5479)	929.4–8780	3650 (2802, 5738)	887.1–8653	0.9901

**Table 5:** Spearman correlation coefficients among urinary biomarkers of oxidative stress in acute exposure (U6); a: Spearman correlation coefficient, b: * (*p* < 0.05), ** (*p* < 0.01), and *** (*p* < 0.001).

	8-Isoprostane		8-OHdG		8-OHG		o-Tyrosine		5-OHMeU		HNE	
8-Isoprostane			0.535	*	0.315	*	−0.252		0.066		0.594	**
8-OHdG	0.535 ^a^	* ^b^			0.723	***	−0.049		0.075		0.446	*
8-OHG	0.315		0.723	***			0.088		−0.049		0.380	
o-Tyrosine	−0.252		−0.049		0.088				0.593	*	−0.198	
5-OHMeU	0.066		0.075		−0.049		0.593	**			−0.041	
HNE	0.594	**	0.446	*	0.380		−0.198		−0.041			

**Table 6:** Comparison of urinary OS markers with the literature values. Units for all biomarkers have been converted as needed and expressed in ng analyte/mg creatinine (or μg/g). Table captures relevant studies for comparison purposes and is not intended to be a comprehensive review.

Biomarker	Study Cohort	AM ± SD	Median	Range or Max	Values in the Current Study Relative to Others
8OHdG					
Zhang et al. 2022 (This study)	Volunteers, U6 copier operators, chronic	5.45 8.11	5.4 5.67	2.63–9.2 2.62–36.18	
Wu et al. 2019 [42]	Workers exposed to carbon nanotubes and metal oxide (CNTs/MeOx) nanoparticles in Taiwan	F: 43.9 ± 42.1 M: 29.6 ± 24.5	-	-	AM is much lower (~4× than CNT/MeOx workers 99% of values smaller than mean of CNT/MeOx workers Maximum value is comparable
Khatri et al. 2017 [19]	Copier operators, chronic exposures (same samples as this study)	Cntr: 6.82 Chronic: 18.36	-	2.97–15.3 11.36–29.9	AM is ~2× higher than controls AM is 1.3× lower than exposed workers Maximum values is 1.17× lower
Khatri et al. 2013 [18]	Healthy volunteers, single 6-h acute exposure to CENPs (same samples as this study)	Cntr: 6.42 Acute: 13.46	-	2.97–15.3 2.97–27.1	AM is slightly lower than controls AM is comparable Maximum value is 1.06× lower
Buonaurio et al. 2020 [43]	Workers in TiO_2_ manufacturing; also compared to controls and other occupational cohorts	TiO_2_: 19.69 ± 14.0 Cntr: 14.66 ± 6.73 Other cohorts: range of means 4.07–27.80	15.29 Cntr: 13.30	9.99–48.79 Cntr: 6.31–26.88	Most of the results are comparable to the 5th percentile of TiO_2_ workers and TiO_2_ controls, but higher than fiberglass workers. Maximum value is 1.9× lower
Graille et al. 2020 [44]	Review and meta-analysis of background 8-OHdG values in healthy populations by chemical methods	4.0 Range of means in several studies: 2.5–6		IQ: 25–75%: 3–5.5	AM is ~3.5× higher than grand mean of 4 in general population Maximum much higher than 75th percentile of grand normal range
Pelclova et al. 2020 [45]	Workers manufacturing and processing advanced nanocomposites	0.274			AM is 50× higher than in nanocomposite workers
8OHG					
(This study)	Volunteers, U6 Copier operators, chronic	5.44 4.84	5.45 4.69	1.96–9.39 1.38–11.81	
Buonaurio et al. 2020 [43]	TiO_2_ exposed workers	16.02 ± 9.64	14.06	5.50–33.15	Most of the results comparable to the 5th percentile of TiO_2_ workers and controls. Maximum value is comparable to the 95th percentile
	Control	8.89 ± 3.88	7.65	5.50–15.79	AM is comparable to controls
	Other worker cohorts	Range of means: 10.63–34	-	-	AM is comparable to painters and gasoline attendants, but much lower than fiberglass and TiO_2_ workers.
Pelclova et al. 2020 [45]		0.486			AM is 11.9× higher than in nanocomposite workers
5OHMeU					
This study	Volunteers, U6 copier operators, chronic	114 45	78 21	15–123 14–113	
Faure et al. 1996 [46]	Cancer patients before chemotherapy Cancer patients after chemotherapy	6.98 ± 0.426 8.05 ± 0.54		7.2–12.2 nmol 5-HMUra/mmol creatinine	AM is 10.6× higher than in cancer patients
Pelclova et al. 2020 [45]	Workers manufacturing and processing advanced nanocomposites	0.057			Values are over 550 times higher in nanocomposite workers
HNE					
This study	Volunteers, U6 copier operators, chronic	1.56 3.87	1.17 2.91	0.23–3.07 0.53–10.7	
8-Isoprostane					
This study	Volunteers, U6 copier operators, chronic	0.43 0.96	0.36 0.64	0.16–0.83 0.17–2.77	
Pelclova et al. 2020 [45]	Workers manufacturing and processing advanced nanocomposites	0.035		0.51	AM is 23× higher than in nanocomposite workers. Maximum is ~5.3× higher
Sakano et al. 2009 [47]	Healthy Japanese people	0.74 ± 0.03			AM is 1.1× higher than healthy Japanese people
Lai et al. 2016 [48]	Welders’ pre-exposure to PM2.5 Welders in shipyards exposed to PM2.5	37 51			AM is 28× lower than welders’ pre-exposure, 34× lower than welders’ post-exposure
O-Tyrosine					
This study	Volunteers, U6 copier operators, chronic	96.56 134	38 52	4.89–100 9.2–115	
Pelclova et al. 2020 [45]	Workers manufacturing and processing advanced nanocomposites	0.265	-		AM is 20× higher than in nanocomposite workers

**Figure A1:**
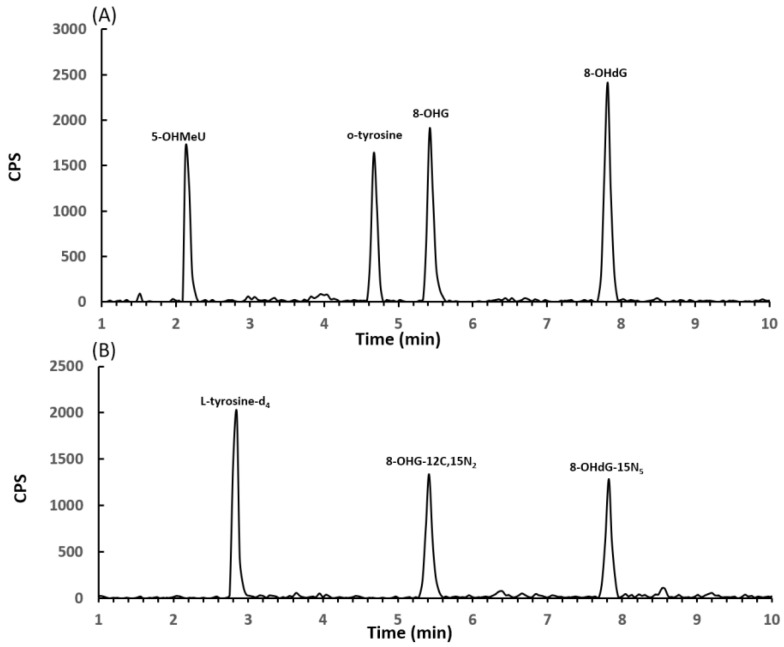
(**A**) A typical LC-ESI-MS/MS chromatogram of a urine sample from the current study participants, containing quantifiable amounts of biomarkers of DNA/RNA damage (5-OHMeU, 8-OHG, 8-OHdG) and protein oxidation (o-tyrosine); (**B**) Chromatogram of their corresponding internal standards in the sample.

**Figure A2:**
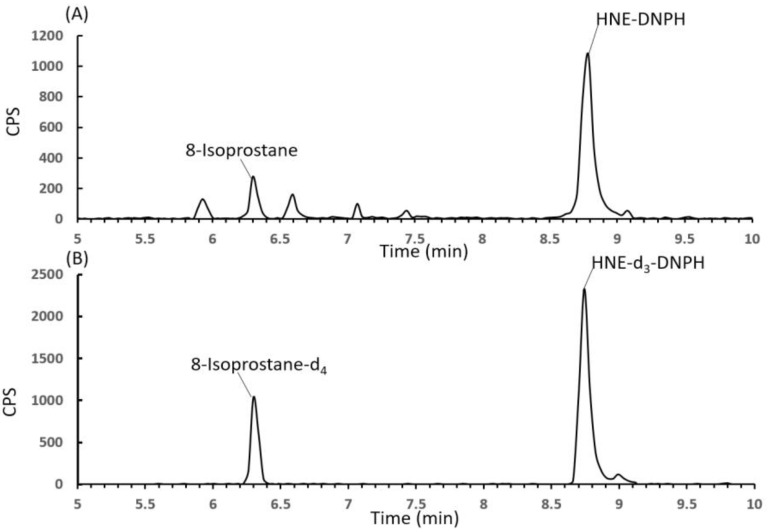
(**A**) A representative chromatogram of a urine sample analyzed for lipid oxidation markers; (**B**) The chromatogram of their corresponding internal standards in the sample.

## Data Availability

The data is available upon reasonable request by emailing the corresponding authors.

## Appendix A

LC-ESI-MS/MS analysis: Apparatus and conditions.

(1) MS/MS acquisition method

All urine samples were analyzed using a Shimadzu LC-20AD chromatographic system coupled to an API 3200 triple quadruple mass spectrometer that was equipped with a Turbo Ion Spray sourced (Applied Biosystems, Foster City, CA, USA). Fraction A, containing 8-OHdG, 8-OHG, o-tyrosine, and 5-OHMeU, were analyzed in the positive electrospray ionization mode (ESI+) with nitrogen gas as the nebulizing (gas 1), heater (gas 2), and curtain gas. The ESI parameters and gas flow were optimized as follows: the ion spray voltage was at 5500 V, the temperature of the ion spray was set at 600 °C, the nebulizer at 65 psi, the heater at 50 psi, and the curtain gas at 30 psi.

Fractions B (8-isoprostane and analogues) and C (HNE-DNPH derivative) were analyzed in the negative ionization mode (ESI^−^), with the ion spray voltage set at −4500 V and ion spray temperature at 600 °C. The gas flow parameters were the same as for Fraction A. The analytes and their corresponding isotopically labeled internal standards were measured in the multiple reaction monitor mode (MRM). The MRM transition and MRM parameters are summarized in Table A1 and include the following: declustering potential, entrance potential, collision energy, collision cell entrance potential, collision cell exit potential, and specific MRM transition for each analyte.

(2) Chromatographic method:

For Fraction A, a Kinetex phenyl-hexyl column (100 mm × 4.6 mm, 2.6 µm particle size) was used. The mobile phase A was 0.1% formic acid in water and mobile phase B was 0.1% formic acid in methanol. Column temperature set at 40 °C. A gradient elution was applied at a flow rate of 600 µL/min: 1% B for the first 2 min, to 7% B at 10 min, in a linear gradient, followed by column re-equilibration at 1% B for 3 min. The sample injection volume was 10 µL.

For Fractions B and C, chromatographic separation was achieved on a Kinetex C18 column, (100 mm× 4.6 mm, 2.6 μm particle size) (Phenomenex, Torrance, CA) at a flow rate of 600 μL/min, with a column temperature set at 40 °C. The mobile phase A was 0.1% ammonium hydroxide in water and mobile phase B was 0.1% ammonium hydroxide in methanol. The gradient elution was as follows: 25% B for the first 2 min, to 90% B at 12 min, in a linear gradient, followed by column re-equilibration for 3 min at 25% B. Sample injection volume was 10 µL.

Figure A1 shows a typical chromatogram of a urine sample from the current study participants, containing quantifiable amounts of biomarkers of DNA/RNA damage. Figure A2 shows a typical chromatogram of a urine sample from the current study participants, containing quantifiable amounts for biomarkers of lipid peroxidation.

(3) Method validation:

The LC-ESI-MS/MS method for analysis of biomarkers of oxidative stress was validated by assessing the following parameters: accuracy, precision, sensitivity, recovery, calibration curve performance, and process efficiency. Calibration curve standards were prepared by spiking the OS biomarker stock solution into synthetic urine (surrogate matrix blank) over the whole linear range (Table 1). In order to obtain the quality control (QC) samples, three concentrations (1 ng/mL, 10 ng/mL and 100 ng/mL) of standards were spiked into the surrogate matrix blank. Each of the three spike concentrations of QC samples were prepared in 5 replicates (n = 5). All calibration standard samples and QC samples were processed in the same way as the urine samples.

The accuracy was evaluated based on the percent of the determined concentration of QC samples relative to the nominal concentration. Accuracy was reported as relative error (RE). The precision was determined by calculating the relative standard deviation of replicates with on sample run (intra-day) and between sample runs (inter-day).

The calibration curve performance was evaluated based on its slope, intercept, and correlation coefficient (r^2^).

The limit of detection (LOD) and limit of quantification (LOQ) were determined based on the instrument response with the integrated function of the Analyst 1.4.2 software (Applied Biosystems). These calculations were based on the signal/noise ratios of 3 and 10 for LOD and LOQ, respectively. The recovery was expressed as the ratio of the observed concentration of QC samples to the nominal concentration.

The matrix effect was investigated by comparing the calibration curve of OS biomarkers in the surrogate urine matrix against the calibration curve in methanol. In this study, these two calibration curves had similar slopes, indicating that no significant matrix effect was present. The regression coefficient of the standard calibration curve in methanol was nearly identical (and statistically not significant) to that of the standard calibration curve in surrogate urine matrix. This should not come as a surprise since we engaged in extensive sample preparation protocol using solid-phase extraction cleanup and fractionation; and excellent chromatographic separation on two distinct analytical columns and ionization modes. The combined result of this front-end analytical work was excellent sample purification and chromatographic separation (Figure A1 and Figure A2), which minimized the likelihood of the co-elution of interfering peaks and urine matrix effects.

The process efficiency is the yield of analytes in a sample preparation process. The QA samples were subjected to SPE treatment. Unlike previous QA samples, the mixture of internal standards was added post SPE. The process efficiency was calculated from the ratio of the observed concentration on these QC samples to the nominal concentration.

**Table A1:** Multiple Reaction Monitoring (MRM) transitions and the optimized compound-specific source parameters for biomarkers and their corresponding internal standards.

Compound Name	MW (g/mol)	MRM Transition	Deculstering Potential (V)	Entrance Potential (V)	Collision Energy (eV)	CEP (V)	CXP (V)
8-OHdG	283	284/168	24.47	4.1	21.18	18.09	3.0
8-OHdG-15N_5_ (IS)	288	289/173	25.52	4.0	22.05	18.25	3.0
8-OHG	299	300/168	27.06	4.92	25.98	18.68	4.0
8-OHG-13C,15N_2_ (IS)	302	303/171	28.00	5.00	27.00	18.68	4.0
5-OHMeU	142	143/125	27.00	5.00	15.00	10.00	3.00
8-Isoprostane	354	353/193	−79.94	−6.93	−37.4	−15.00	16.4
8-Isoprostane-d_4_ (IS)	358	357/197	−76.94	−7.50	−38.5	−15.00	16.4
O-Tyrosine	181	182/136	40.00	5.00	19.46	14.845	5.00
L-Tyrosine-d_4_ (IS)	185	186/140	42.25	5.25	18.52	14.50	5.00
HNE-DNPH	336	334.9/182	−50.2	−4.5	−32	−18.1	−16
HNE-d_3_-DNPH (IS)	339	337.9/182	−52.3	−4.5	−31	−15.6	−16

**Table A2:** Retention times and calibration curves for the panel of biomarkers of oxidation of DNA/RNA, lipids and proteins.

Marker	Compound Name	Retention Time (min)	Slope	Intercept	R^2^	*p* Value for Intercept
DNA & RNA damage	8-OHdG	7.83	0.1744	0.7458	≥0.9992	0.069
	8-OHG	5.41	0.285	0.2652	≥0.9989	0.37
	5-OHMeU	2.12	0.0126	0	≥0.9961	0.082
Protein oxidation	O-Tyrosine	4.69	0.4101	−0.8879	≥0.9991	0.055

Lipid peroxidation	HNE-DNPH	8.89	0.1997	−0.0397	≥0.9991	0.72
	8-Isoprostane	6.24	0.1796	−1.8095	≥0.9992	0.0076
